# Seasonal and inter-annual dynamics in the energy seascapes of a marine top predator

**DOI:** 10.1098/rsos.250929

**Published:** 2025-10-22

**Authors:** Aurélien Favreau, Jérôme Spitz, Martin Huret, Guillermo Boyra, Mathieu Doray

**Affiliations:** ^1^ DECOD, L'Institut Agro, IFREMER, INRAE, Plouzané, France; ^2^ Observatoire Pelagis, UAR 3462, La Rochelle Université/CNRS, 17000 La Rochelle, Nouvelle-Aquitaine, France; ^3^ AZTI, Instituto Technologico Pesquero y Alimentario, 20110 Pasaia, Spain; ^4^ DECOD, L'Institut Agro, IFREMER, INRAE, Nantes, France

**Keywords:** common dolphin, energy seascape, small pelagic fish, Bay of Biscay

## Abstract

Ecosystems are undergoing global changes that disrupt energy transfer across trophic levels, reducing energy availability and destabilizing predator–prey interactions. In marine systems, small pelagic fish (SPF) are key energy vectors to higher trophic levels. In the Bay of Biscay, common dolphins rely heavily on SPF, which have declined in size and condition in recent decades, while dolphin by-catch has increased. To explore whether prey field changes could explain shifts in dolphin distribution, we mapped prey energy by integrating biomass, size and energy content for four SPF species (sardine, anchovy, sprat and small horse mackerel), representing 80% of the dolphin diet. We analysed acoustic survey data from PELGAS (2000–2023) and JUVENA (2009–2023) to assess temporal and spatial changes in prey energy. Energy per prey has declined, mainly due to decreasing fish length and energy density. Total energy available for common dolphin remained stable, through increase of lower-energy anchovies and decrease of larger, higher-energy sardines. Spatial redistributions were also evident, with sardine energy becoming more coastal and anchovy energy expanding northward. These energy seascapes dynamics, probably climate-driven, may have led dolphins to forage closer to shore, in areas of higher fishing pressure, thereby contributing to the recent rise in by-catch.

## 1. Introduction

In the context of global change, ecosystems are undergoing profound transformations that disrupt energy transfer processes across trophic levels [1–3]. These disruptions can reduce the overall energy available within food webs, thereby affecting ecosystem structure and functioning [4]. In marine ecosystems, reduced energy transfer efficiency may result from various processes, including changes in primary productivity, shifts in species distribution and alterations in prey availability and quality [5,6]. These changes can destabilize trophic interactions through bottom-up or cascading effects, ultimately threatening food-web integrity [7,8]. One step towards understanding how these disruptions propagate through the marine food web is to examine both the dynamics of mid-trophic level species—such as small pelagic fish—and the responses of their predators. Top predators are particularly vulnerable to changes in prey fields [9,10], as they depend on the efficient transfer of energy from lower trophic levels to sustain key biological functions like growth, reproduction and survival [11]. As such, their condition can provide valuable insight into broader ecosystem changes [9].

The optimal foraging theory (OFT) [12–14] provides a framework for interpreting predator responses to environmental changes. According to OFT, predators aim to maximize the ratio of energy gained from a prey to the energy spent in searching for and capturing it. While prey quantity has long been considered a key factor in foraging success, prey quality has emerged as an equally critical factor in understanding predator behaviour [15,16]. Prey quality, often represented by energy density—the amount of energy per unit of mass—plays a central role in foraging efficiency [17]. A decrease in prey energy density can significantly reduce the total energy available to predators, even if prey abundance remains constant [17,18]. Rosen and Trites [19] showed that Steller sea lions are unable to fully compensate for such a decline by simply increasing prey consumption due to digestive limit. Prey size is also a key component of prey quality as larger individuals contain more energy than smaller ones at a given energy density.

The concept of ‘energy landscape’ [20] has extended OFT by integrating the spatial dimension of foraging in an heterogeneous environment. Within this framework, foraging costs are often calculated based on energetic costs associated with different variables such as travel duration, diving duration and depth or swimming speed [20–24]. The resulting landscapes (or seascapes thereafter) represent maps of energy intake and/or expenditure. Such maps have been primarily developed in the marine realm for seabirds, fish or pinnipeds, facilitated by the recent advances and widespread use of electronic devices such as biologging tags [25,26]. These devices have enabled detailed tracking of animal movements and behaviour, allowing researchers to infer energy landscapes. However, mapping energy seascapes of some species of small cetaceans such as the common dolphin remains challenging, due to logistical difficulties to equip such fast-moving, free-ranging species with biologgers [27,28]. Small cetaceans are also more vulnerable to injury from tagging, and common dolphin are furthermore highly sensitive to stress, making any handling attempt risky.

Common dolphin *Delphinus delphis* [29] is one of the most widely distributed and abundant cetaceans worldwide [29,30]. In the Bay of Biscay (BoB hereafter), it is abundant in both neritic and oceanic habitats [31]. This small-sized dolphin displays active swimming behaviour, resulting in high metabolic cost and substantial energy requirements [32]. Common dolphins are specialist predators foraging on high-energy prey like myctophid fish in oceanic waters [33] and small epipelagic fish in neritic areas [17,34,35]. While the specific composition of their diet may vary across years and seasons [34], they consistently switch from one high-energy prey to another. For example, following a decline in horse mackerel, their consumption of anchovy, has increased [36]. Conversely, they tend to avoid low-energy prey species such as gadoids, even when these are abundant in their feeding grounds [17,37]. This behaviour confirms that prey quality is at least as important as prey quantity for common dolphins, as for other species [15,38].

Small pelagic fish (SPF) with high fat and energy content play a major role in energy transfer from lower to higher trophic levels [39–41]. In the BoB, key neritic prey of common dolphins include European anchovy *Engraulis encrasicolus* [29], hereafter anchovy, European sardine *Sardina pilchardus*, hereafter sardine, Atlantic horse mackerel *Trachurus trachurus* [29], hereafter horse mackerel, and European sprat *Sprattus sprattus* [29], hereafter sprat [34]. Over the past two decades, these species have experienced substantial fluctuations in abundance [42]. In the meantime, a marked decrease of anchovy and sardine length and body condition has also been observed in the BoB [43–45]. This decline in fish condition can have implications for energy transfer to top predators. Additionally, the displacement of sardine spring spawning grounds closer to the coast [46] and the northward expansion of anchovy [47] may have further influenced the distribution of their predators. Moreover, seasonality is known to play a major role in shaping SPF distribution [47–49] and to influence their energy dynamics. Individuals are in poorer condition after the winter and replenish their energy reserves by taking advantage of favourable feeding conditions from spring to autumn in the BoB [50,51]. These pronounced seasonal differences in energy availability probably play a key role in shaping predator foraging behaviour and broader ecological dynamics in the BoB [52]. A better understanding of these ecological dynamics is crucial, to ensure the conservation of the threatened BoB common dolphin population.

Common dolphin has been by-caught by fishers for decades in the BoB [53]. But since 2016, the number of stranded dolphins showing evidence of by-catch has risen dramatically, peaking at 1200 individuals on the French coast in 2019 [54]. Despite this important rise in by-catch rates, no significant increase in total fishing effort has been documented to explain the surge in dolphin by-catch [55]. However, aerial sighting surveys conducted in 2021 have revealed a shift in the common dolphin distribution in the European Atlantic area, where dolphins were more dispersed over both continental shelf and oceanic habitats in summer and winter compared with 2012 [55]. It has been assumed that changes in common dolphin distribution might be an adaptation to changes in their epipelagic prey’s energy seascapes, as observed in other marine predators. Similar patterns have been observed in other marine predators such as bluefin tunas that shifted their distribution following declines in herring mean weight [16].

To test the presence of a shift in dolphin energy seascapes in the BoB, while assessing their seasonal variations, we characterized the energy distribution of its small pelagic prey community during spring (2000−2023) and autumn (2009−2023), based on spatial distribution, abundance and energy content derived from scientific surveys. We analysed the space-time variations of these energy seascapes considering the total and individual energy at the population and community levels of SPF.

## 2. Material and methods

### 2.1. Study area and data

**Figure 1.**
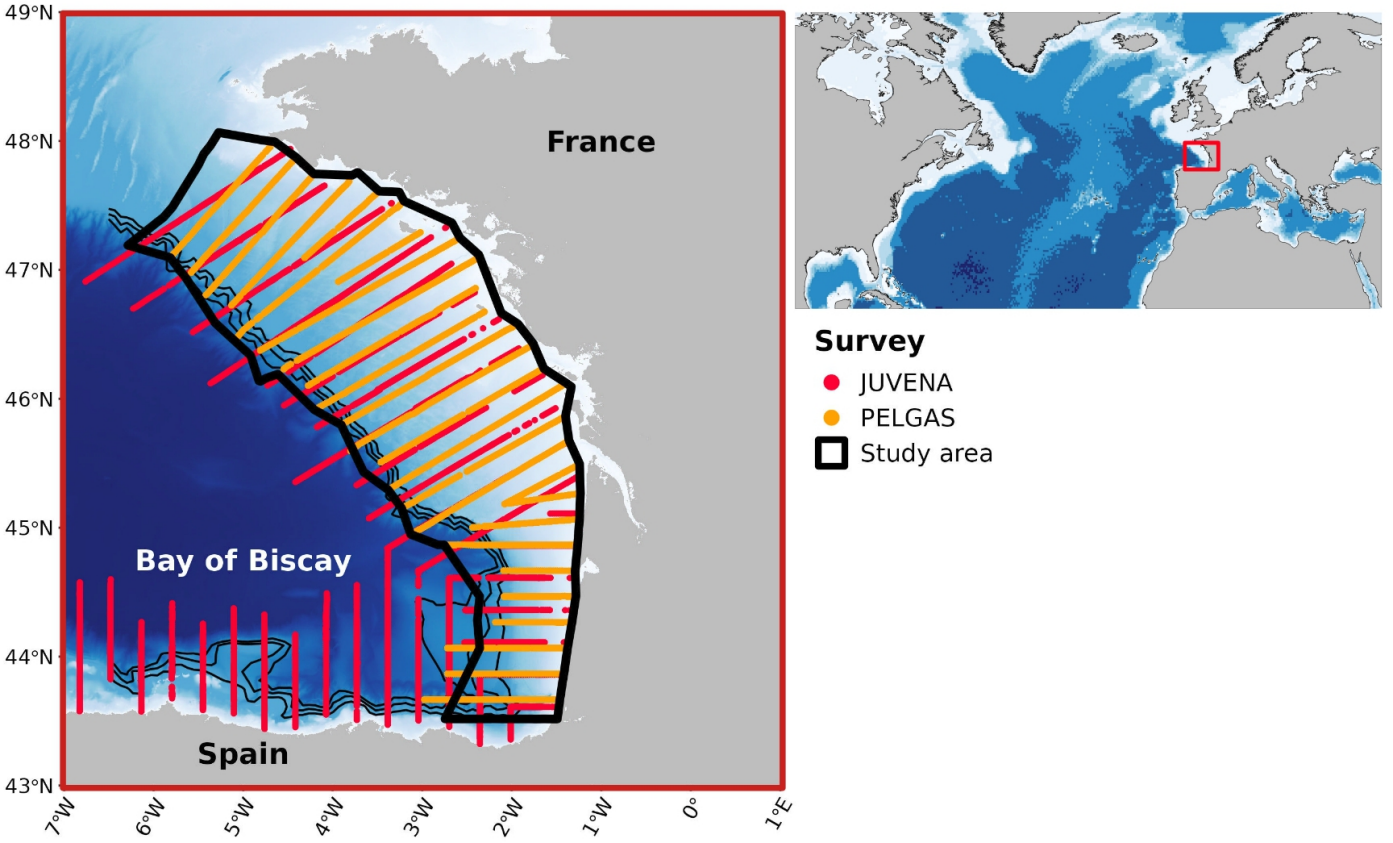
Acoustic transects of the annual PELGAS (spring) and JUVENA (autumn) integrated surveys in the BoB. Black polygon: study area; black lines: 500, 1000 and 1500 m isobaths, marking the edge of the continental shelf.

The BoB is an open oceanic bay delimited by the French coast in the eastern part and the Spanish coast in the southern part (figure 1). Two integrated acoustic surveys have consistently covered the BoB on an annual basis: PELGAS in spring (May, since 2000 [56]) and JUVENA in autumn (September, since 2003 [57]). Both surveys used standard protocols to assess SPF abundance based on acoustic-trawl data [58]. PELGAS has been conducted by IFREMER on the RV ‘Thalassa II’ and commercial pair trawlers to monitor small pelagic fish and their ecosystem in spring, while JUVENA has been conducted by AZTI on different vessels, mainly the RV ‘Ramо́n Margalef’ or RV ‘Angeles Alvariño’ and the RV ‘Emma Bardán’, to assess juvenile anchovy in their ecosystem, in early autumn. In this study, we used PELGAS survey data collected from 2000 to 2023, with a gap in 2020 due to the COVID-19 pandemic. We used JUVENA data collected from 2009 to 2023 while excluding 2019, to ensure consistency in the spatial coverage between both surveys.

### 2.2. Biomass and abundance of dolphin’s prey

In BoB neritic waters, Meynier et al. [34] identified sardine, anchovy, sprat and horse mackerel from 8 to 25 cm long as the most significant species in the fresh diet of common dolphin, (representing 44.9, 22.6, 8 and 5% in mass, respectively). They represented approximately 80% of common dolphin diet in the BoB. Those species and size range were then selected to represent dolphin prey in this study. Acoustic data were collected during daytime along systematic line transects perpendicular to isobaths (figure 1), spaced 12 nautical miles (nm) away for PELGAS and 15 nm away for JUVENA with elementary distance sampling unit (EDSU) set to 1 nm for PELGAS and 0.1 nm for JUVENA. The PELGAS survey has annually covered the BoB French continental shelf, whereas JUVENA survey has covered the French and Spanish northeastern BoB continental shelf as well as offshore waters (figure 1). JUVENA coverage has varied annually to adaptively cover the distribution of anchovy juveniles. Here, we only considered the continental shelf area consistently covered by both surveys along the series (figure 1). Fish acoustic densities have been combined with trawl catches to estimate biomass, abundance, mean weight and mean length per EDSU for all SPF species in both surveys following a standard procedure [58].

### 2.3. Energy density

Energy density (ED) values of 333 sardines, 197 anchovies, 34 sprats and 25 horse mackerel in spring, and 135 sardines, 157 anchovies, 32 sprats and 67 horse mackerel in autumn were measured in the BoB between 2014 and 2018 using bomb calorimetry. For sardine and anchovy, data are available in [59]. A generalized additive model (GAM) was applied to estimate fish energy density ED as a function of fish length *L* and season *S* for each species,


(2.1) $$ED_{L,S}=\beta_{0}+s\left(L,S\right)+\beta_{1}S+\epsilon ,$$

where *β*_0_ is the intercept, *s*(*L*,*S*) represents the smoothing function of length for each season, *β*_1_*S* accounts for a fixed seasonal effect and *ϵ* is the residual error, assumed to be normally distributed with mean 0 and constant variance. Given that ED was not routinely monitored during scientific surveys [51], we assumed that the relationship between ED and length per season for each species was constant from 2000 to 2023.

### 2.4. Prey energy calculation

We quantified prey energy using two metrics: (i) individual energy, corresponding to the energy content (kJ) of an average single prey individual, and (ii) total energy, corresponding to the combined energy of all prey individuals of a given area. The calculation was performed in two steps: first at the EDSU scale, and then aggregated to the BoB scale to capture broader ecological patterns. At this larger scale, metrics were calculated at both the population level (each prey species separately) and the community level (all four species combined).

#### 2.4.1. Energy calculation per elementary distance sampling unit

At the EDSU scale, we first calculated the individual energy *E*_*i*,*s*,*e*_ of an individual *i*, species *s* within an EDSU *e* using equation (2.2),


(2.2) $$E_{i,s,e}=W_{i,s,e}.ED_{l,s,p},$$

where *W*_*i*,*s*,*e*_ is the mean weight of individual *i* of species *s* within EDSU *e*, and *ED*_*l*,*s*,*p*_ the energy density for a fish of length *l*, for species *s* in season *p*.

Total energy per species and EDSU *E*_*s*,*e*_, was then obtained by multiplying individual energy by species abundance *A*_*s*,*e*_ in that EDSU (equation (2.3)),


(2.3) $$E_{s,e}=E_{i,s,e}.A_{s,e}$$

This step provided georeferenced estimates of total energy for sardine, anchovy, sprat and horse mackerel, by combining acoustic-trawl survey indices with energy density estimates.

#### 2.4.2. Individual and total prey energy at the scale of the Bay of Biscay

EDSU-level calculations were required to construct energy seascapes, but BoB-scale estimates are more relevant from an ecological perspective. Individual energy (in kJ) was calculated as the ratio of total energy over total abundance summed over all EDSU *e*. At the population level, this corresponds to the average energy of an individual prey of species *s* in year *y*. At the community level, this corresponds to the average energy of an individual prey across all species combined in year *y*. These two metrics were calculated as follows:


(2.4) $$E_{s,y,i}=\frac{\sum_{e}E_{s,y,e}}{\sum_{e}A_{s,y,e}}\;\text{and}\;E_{y,i}=\frac{\sum_{e,s}E_{s,y,e}}{\sum_{e,s}A_{s,y,e}},$$

where *E*_*s*,*y*,*e*_ and *A*_*s*,*y*,*e*_ are, respectively, the total energy and total abundance of species *s* in year y and EDSU *e*.

To facilitate spatial analysis, calculations of total energy were performed on a regular grid (described in the following section). For each year *y* and prey species *s*, we calculated the total available energy in the BoB *E*_*s*,*y*_ as the sum over grid cells *g* of the product of the energy content by the area of the BoB. At the community level (*E*_*y*_), this process was repeated using all species combined,


(2.5) $$E_{s,y}=\sum_{g}E_{s,g,y}\;\text{and}\;E_{y}=\sum_{g,s}E_{s,g,y}.$$

### 2.5. Statistical analysis

#### 2.5.1. Average energy seascapes

Georeferenced energy data were averaged using a block averaging method [60] over a standard grid across all years. A grid mesh size of 0.25° (approx. 15 km) was selected to interpolate per-EDSU results on the grid, while ensuring that enough samples (approx. 10) were averaged within each grid cell for each year, to ensure the highest spatial resolution [61]. This spatial smoothing process provided annual gridded maps representing the average energy seascapes (average energy available in kJ) for common dolphins from 2000 to 2023 in spring, and from 2009 to 2023 in autumn. We further investigated the space-time variations of these average prey energy seascapes at the individual, population and community levels. Here, the term population refers specifically to all individuals of the same species in the BoB, even though the actual population may extend beyond this area.

#### 2.5.2. Effects of fish length and energy density on individual prey energy

We so far assumed that the SPF energy density was constant. Variations in individual prey energy hence only reflected changes in fish length (or weight) for a given species in this baseline scenario. However, a decrease of sardine body condition has been reported in the BoB [44]. It was corroborated by the concurrent increase of sardine water content routinely measured by the canning industry as a fish quality index (https://defipel-tdb.ifremer.fr/), which suggests a decline in fat content or ED [51]. To evaluate the potential impact of this decline in ED relatively to fish length decrease, we built two alternative scenarios: (1) decreasing ED with constant length; and (2) decreasing ED combined with observed decreasing length. The decline in ED for each species and season was estimated over the period for which survey data were available in spring and autumn (2009–2023). It was calculated based on water content measured by the sardine canning industry (2007−2023), using the methodology of [51], which accounts for fish condition state. ED estimates were calculated based on water content data collected in April and May for spring, and October and November for autumn, to match the survey timings. The ED linear decrease was non-significant in spring (model slope *p*‐value = 0.14, electronic supplementary material, figure A1), but was strongly significant in autumn (model slope *p*‐value < 0.05, electronic supplementary material, figure A1). We calculated the percentage change in ED for each year from 2009 to 2023 relative to a reference year with direct ED measurements (2015) and we applied this change to the GAM of energy density as a function of length for both seasons across all four species (sardine, anchovy, sprat and horse mackerel, electronic supplementary material, appendix A, figures A2 and A3) due to the lack of water content time series for species other than sardine. The constant fish length for each species (used in scenario 1) was calculated by determining the abundance-weighted average length per EDSU for each year from 2009 to 2023, and then averaging these yearly values across the survey series. Fish weights for each species, required in equation (2.1), were from these lengths using species-specific length–weight relationships from PELGAS and JUVENA surveys. Finally, the regression slopes of individual energy over time were compared across scenarios to assess the relative impact of length and energy density on fish individual energy.

#### 2.5.3. Space-time variations of energy seascapes

Empirical orthogonal function (EOF [62]) analysis was used to explore space-time variations of common dolphins prey’s energy seascapes in springtime and autumn. EOF is a principal component analysis (PCA) adapted for the space-time analysis of time series of maps [63]. This method decomposes the space-time variability of the anomalies (residuals) around an average map, into principal spatial modes and their associated amplitudes over time. Spatial modes, or EOF maps, characterize the hierarchized patterns of spatial variability in the anomalies. EOF amplitudes time series describe the variations of spatial modes over time [64]. We selected EOF modes that explained more than 20% of the total variance of anomalies and had a local explained variance (percentage of explained variance per grid cell) greater than 50% over at least 20% of the study area. The space-time variability of prey energy was also examined in three dimensions in springtime by integrating two depth strata (above and below 30 m depth) to EOF analysis, as depth-stratified data were available for the PELGAS survey. Time series of the centres of gravity (CG [65]) coordinates of the prey energy gridded maps were also analysed to assess shifts in prey spatial distributions.

## 3. Results

### 3.1. Prey energy density modelling

**Figure 2.**
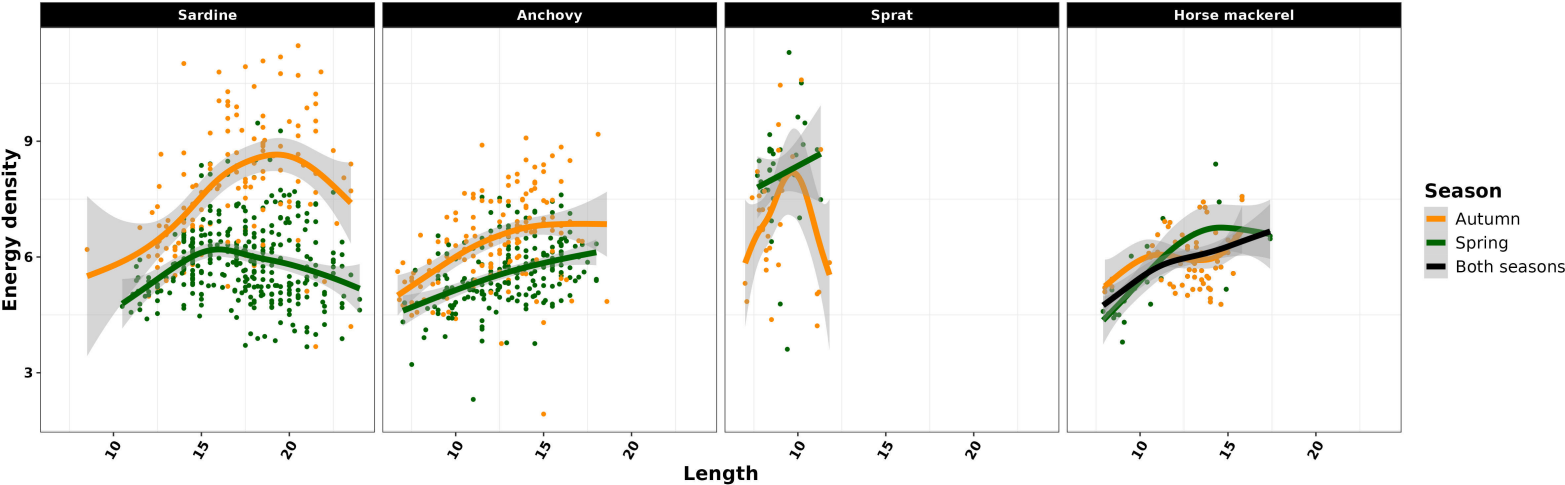
Observed (dots) and predicted (lines) energy density values by the GAM against length (cm) for autumn and spring for sardine, anchovy, sprat and horse mackerel. For horse mackerel, a single GAM including both seasons is represented as the seasonal effect is not significant.

GAM modelling showed a significant effect of length (*p* < 0.05) on energy density for all species and season, except for sprat in autumn (*p* = 0.34) (figure 2, electronic supplementary material, table A1). Additionally, a significant seasonal effect was observed for all species (*p* < 0.05), except horse mackerel (*p* = 0.56), for which the best GAM model had no season effect (figure 2, electronic supplementary material, table A1). Sardine and anchovy energy density increased in autumn, particularly for larger individuals. However, a decline was observed for sardine individuals larger than 20 cm.

### 3.2. Temporal variations of common dolphin prey energy

#### 3.2.1. Individual prey energy

**Figure 3.**
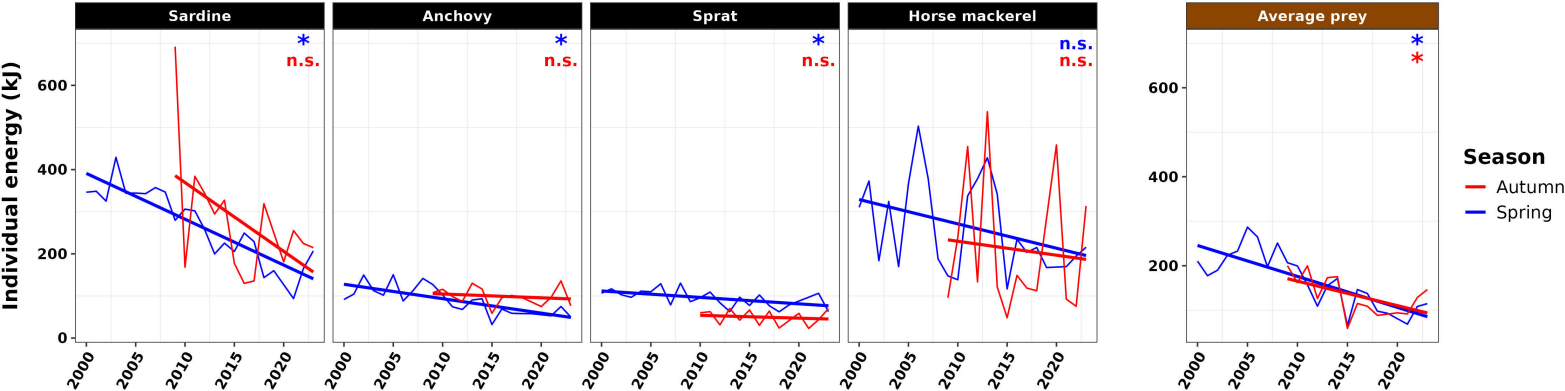
Time series of the individual energy of the prey of common dolphin (in kilojoules, kJ) in the BoB, for an average prey by species (sardine, anchovy, sprat, horse mackerel) and for an average prey of the SPF community, in spring and autumn. Model slope significance level: non-significant (*p*-value > 0.05, noted n.s.), significant (*p*-value < 0.05, noted *).

In our baseline scenario considering only a decrease of prey length, individual energy showed a notable decline throughout the time series in spring in the BoB (figure 3). Energy of sardine and anchovy individuals exhibited significant reductions of approximately 2.5- and 2-fold, respectively, from 2000 to 2023 (*p*‐value < 0.05). In absolute terms, the decline in sardine energy was the most pronounced among the species considered, followed by anchovy. Sprat also showed a significant decrease, albeit with higher variability (*p*‐value < 0.05), while no statistically significant change was observed for horse mackerel.

The energy of the individual average prey declined 2.2-fold, from approximately 220 kJ in the early 2000s to 100 kJ in the 2020s. This trend resulted from the increase of the abundance of smaller, less energetic individuals, such as anchovy.

In autumn, individual prey energy did not show significant trend for any single species over the period 2009−2023. A significant decreasing trend was present in the energy of the average individual prey, reflecting the increasing proportion of smaller, less energy-dense species, such as anchovy, in the BoB ecosystem in autumn.

#### 3.2.2. Total energy at the population and community levels

**Figure 4.**
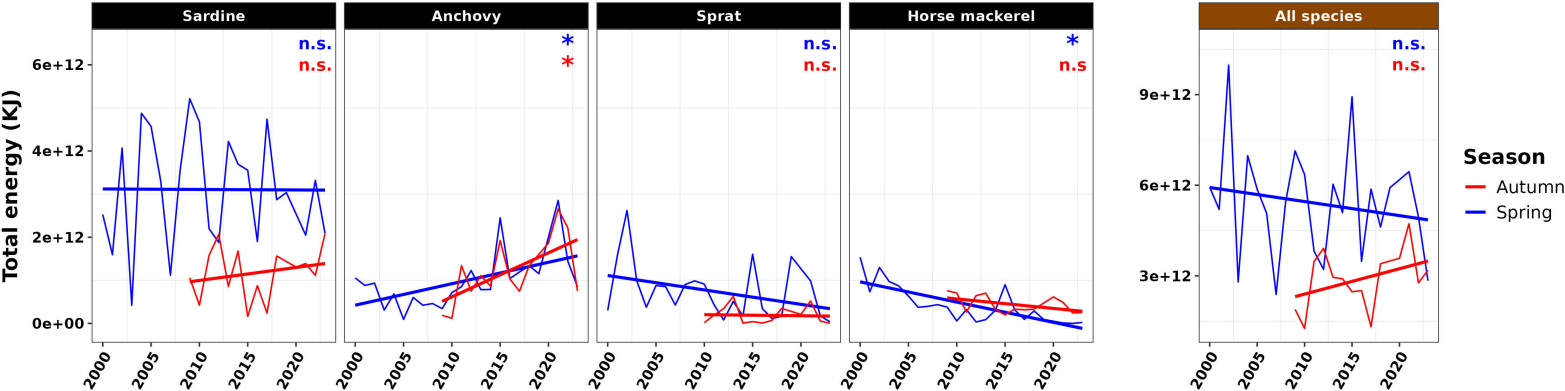
Time series of the total energy of the prey of common dolphin (in kilojoules, kJ) in the BoB, at the population (sardine, anchovy, sprat, horse mackerel) and community (all species) scales, in spring and autumn. Model slope significance levels: non significant (*p*-value > 0.05, noted n.s.), significant (*p*-value < 0.05, noted *).

The total energy available from common dolphin prey varied markedly over time, both at the population and community levels (figure 4). At the community level, mean total energy over the studied period was higher in spring than in autumn (paired *t*‐test, *t* = 3.9, *p* < 0.01), despite species-specific differences. In spring, anchovy population energy showed a significant linear increase over time (model slope *p* < 0.05), whereas horse mackerel energy declined significantly (model slope *p* < 0.05). No significant trends were observed for sardine and sprat. Sardine contributed the most to total prey energy in spring, accounting for an average of 58%.

In autumn, anchovies were again the only species showing a significant increase in population energy over time. No significant trend was detected for sardine, horse mackerel or sprat. Since 2016, anchovy’s contribution to total autumn energy has increased, becoming comparable to that of sardine.

#### 3.2.3. Effect of length and energy density variations on prey energy

**Figure 5.**
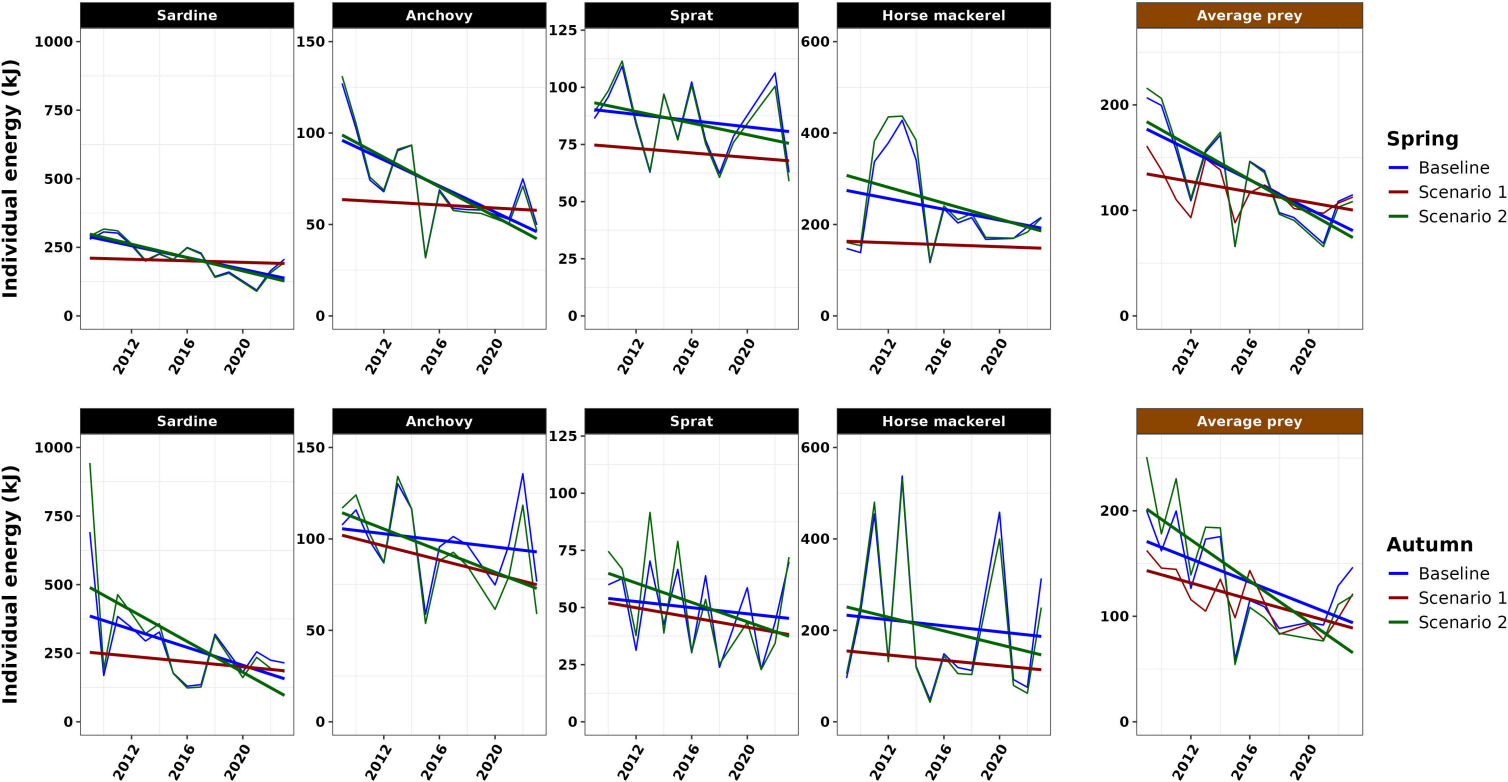
Time series of the individual energy of the prey of common dolphin (kJ) in the BoB, at the population (sardine, anchovy, sprat, horse mackerel) and community (average prey) scales, in both spring (upper panel) and autumn (lower panel), simulated for each scenario from 2009 to 2023. Baseline: constant energy densities (EDs) and variable lengths, scenario 1: decreasing EDs and constant lengths, scenario 2: decreasing EDs and variable lengths.

ED at-length values decreased from 2009 to 2023 in spring and autumn (electronic supplementary material, figures A2 and A3) and were consistent with the variations observed in our data for the three other species, supporting the realism of our scenarios. Individual prey energy estimates for each species, season and scenario are shown in figure 5 (in electronic supplementary material, figure A4 for the spring time series from 2000 to 2023), and regression slopes detailed in table 1. In spring, the baseline scenario regression slopes were steeper than scenario 1 slopes, indicating that length had a stronger effect on individual prey energy than ED. In autumn, the baseline scenario regression slopes were steeper than scenario 1 slopes for sardine, horse mackerel and average prey, indicating that length had a stronger influence on individual prey energy than ED. For anchovy and sprat, regression slopes were steeper for scenario 1, suggesting a larger effect of ED relatively to length on individual prey energy. When combining decreases in both length and ED (scenario 2), the reduction in individual energy was higher relatively to the baseline scenario in spring but more pronounced in autumn. Slopes were generally steeper in autumn than in spring, driven by the larger ED decline in autumn. For the baseline scenario, slopes in spring were steeper than in autumn for all species except sardine. Under scenario 2, slope differences between seasons were less pronounced than for baseline scenario and scenario 1, though sardine exhibited a steeper decline in autumn. Looking at total energy (electronic supplementary material, figure A5), the constant length scenario (scenario 1) had a stronger effect on total available energy in spring than in autumn, due to the lower ED variations in spring. Maintaining a constant length would lead to an increase of total energy at the sardine population and community scales in spring.

**Table 1.** Slope values from linear models assessing the trends in individual energy throughout the years for each scenario and species for both spring and autumn (see figure 5).

species	baseline		scenario 1		scenario 2	
	spring	autumn	spring	autumn	spring	autumn
sardine	−10.74	−20.51	−1.39	−4.79	−12.20	−28.10
anchovy	−3.57	−0.75	−0.42	−1.93	−4.05	−2.96
sprat	−0.68	−0.67	−0.49	−1.07	−1.27	−2.12
horse mackerel	−5.89	−3.31	−1.08	−2.93	−8.68	−7.49
average prey	−6.87	−6.33	−2.45	−3.89	−7.85	−9.73

### 3.3. Average energy seascapes

**Figure 6.**
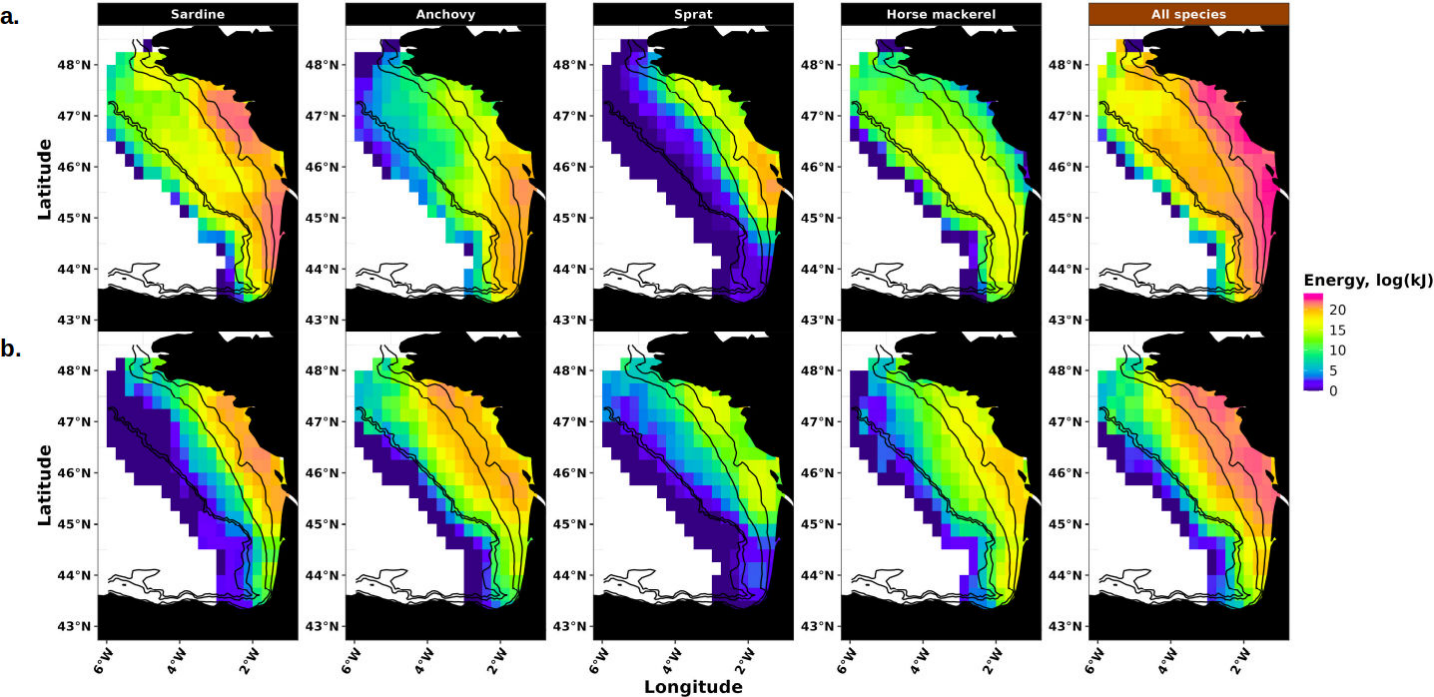
Average energy seascapes of common dolphin. They represent average maps of available prey energy (in log(kJ)) in spring (a) and autumn (b), at the population (sardine, anchovy, sprat and horse mackerel) and community levels (all species). Black lines indicate the 50, 100, 500 and 1000 m isobaths, with the two latter marking the shelf break.

Average energy seascapes of common dolphin in the BoB were derived from annual prey energy maps in spring and autumn (figure 6). Energy distribution patterns varied between species and seasons. Sardine energy was broadly distributed over the BoB in spring, with maximum values along the coast in particular around 45° N and 47° N, as well as over the shelf break. In autumn, its distribution shifted towards the coast, as almost all sardine energy was located within the 100 m isobath and the offshore energy hotspot disappeared. Anchovy energy, was concentrated in the south of the BoB in spring (between 43.5° N and 46° N), and moved northwards in autumn (between 45.5° N and 47.5° N, CG of total energy moved 124 km north). Sprat energy remained near the northern BoB coast during both seasons. Horse mackerel energy was widespread in spring, with a notable offshore component that disappeared in autumn, leading to a more coastal distribution and a core area around 46° N. At the community level, energy was broadly distributed across the BoB in spring, with a coastal core area and a secondary offshore component. However, in autumn, the offshore component disappeared, and energy was primarily concentrated along the coast (between 45.5° N and 47.5° N). Detailed maps showing energy distributions by species, season, and year are available in electronic supplementary material, figures A6–A15.

### 3.4. Space-time variations of common dolphin energy seascapes

#### 3.4.1. Patterns of spatio-temporal variability of the energy

**Figure 7.**
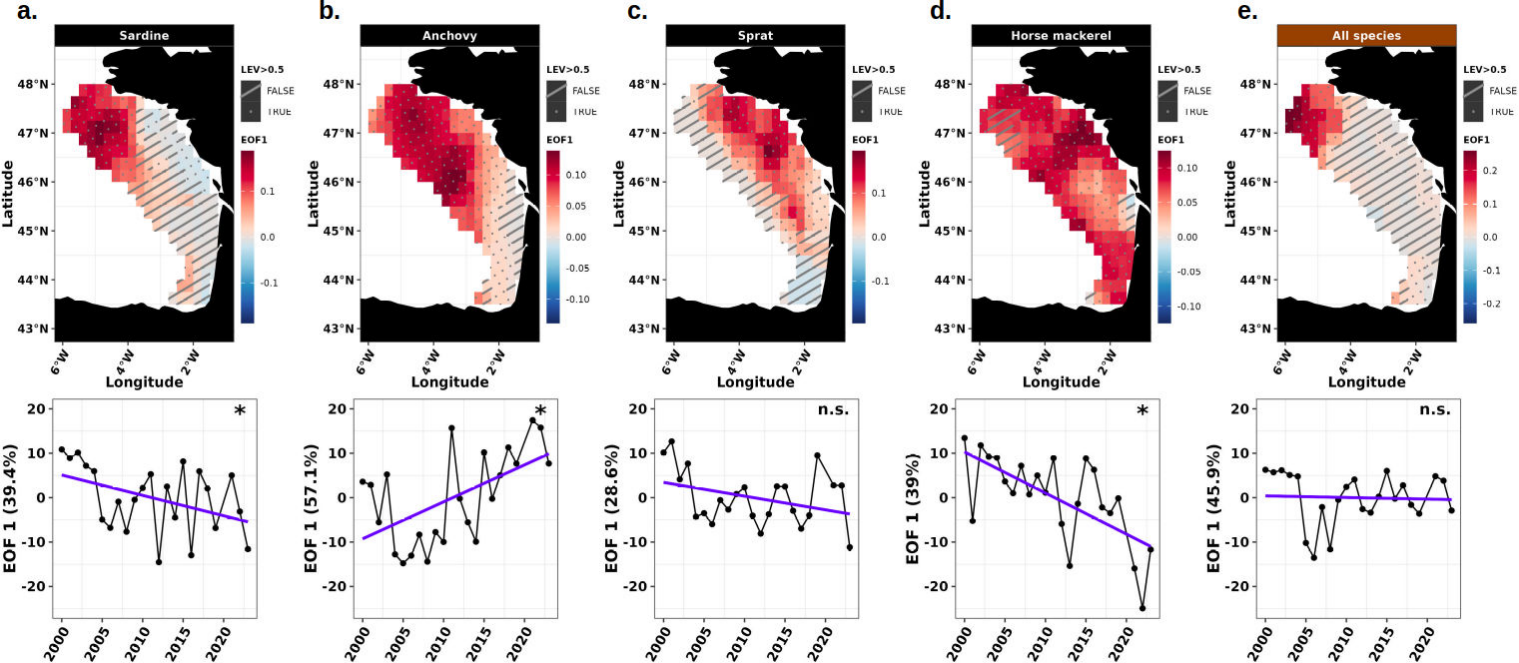
Representation of the EOF analysis of the available energy at the population (sardine, anchovy, sprat, horse mackerel) and community (all species) scales in spring in the BoB. Spatial pattern of the EOF 1 (top panel) and its corresponding amplitude with explained variance (bottom panel) are represented, illustrating the spatio-temporal variation and local explained variance (LEV). Positive EOF anomalies are shown in red, while negative ones appear in blue. Anomalies represent the residuals around the average energy seascape. Local explained variance is indicated by dotted cells when above 50%, and dashed cells when below 50%, where results are considered unreliable for interpretation. Significance level of amplitude slope: non-significant (*p*-value > 0.05, noted n.s.), significant (*p*-value < 0.05, noted *).

**Figure 8.**
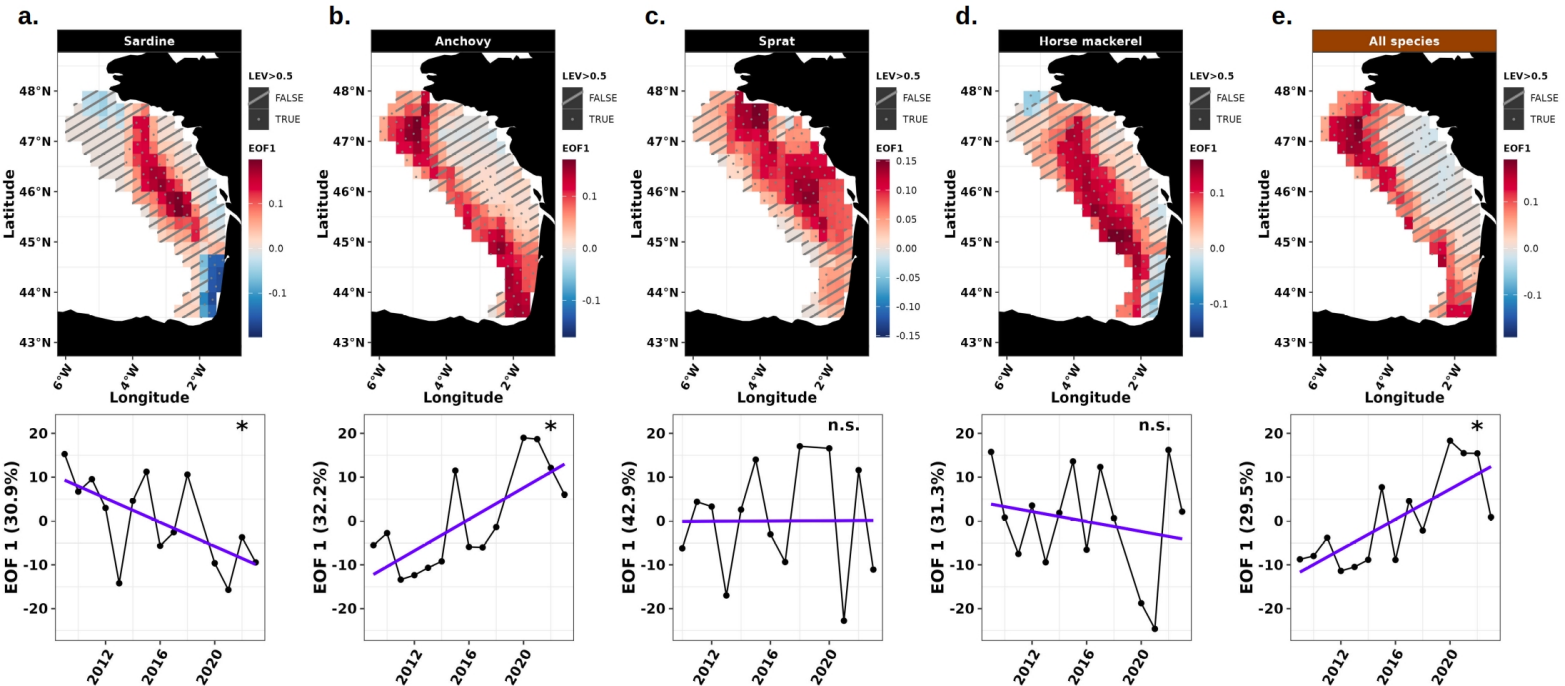
Representation of the EOF analysis of the available energy at the population (sardine, anchovy, sprat, horse mackerel) and community (all species) scales in autumn in the BoB. Spatial pattern of the EOF 1 (top panel) and its corresponding amplitude with explained variance (bottom panel) are represented, illustrating the spatio-temporal variation and local explained variance (LEV). Positive EOF anomalies are shown in red, while negative ones appear in blue. Anomalies represent the residuals around the average energy seascape. Local explained variance is indicated by dotted cells when above 50%, and dashed cells when below 50%, where results are considered unreliable for interpretation. Significance level of amplitude slope: non-significant (*p*-value > 0.05, noted n.s.), significant (*p*-value < 0.05, noted *).

The EOF decomposition method was applied to extract the main spatial modes of variability around the average energy seascape (figure 6) and their associated amplitudes. The first EOF mode and its local explained variance revealed distinct space-time patterns between species in spring (figure 7) and autumn (figure 8). EOF1 captured a substantial portion of the variability in available energy, explaining between 27.7 and 57.1% of the variance in spring, and between 29.5 and 42.9% in autumn at the population and community levels. Higher order EOF modes were not retained as they explained less than 20% of the variability in spring (electronic supplementary material, figure A16) and in autumn (electronic supplementary material, figure A17), and less than 50% of local variance over less than 20% of the study area. For sardine, the first spatial mode (EOF1) explained 39.4% of energy variability in spring, with positive anomalies in the northwestern BoB (figure 7, top panel). This pattern showed a significant decrease until 2005, then stabilized with high interannual variability (figure 7, bottom panel). EOF analyses per depth strata (electronic supplementary material, figures A19 and A20) provided additional insights into three-dimensional space-time variations of sardine energy. In the surface layer, EOF1 explained 27.1% of the variance, with positive anomalies in the northwestern BoB and a significant trend over time. Conversely, in the bottom layer, the EOF1 accounted for 35.5% of the variance, with positive anomalies along the central and northern shelf break with a significant decreasing trend over time. In autumn, EOF1 accounted for 30.9% of the variance, with positive anomalies in the central BoB and negative ones in the southeast (figure 8, top panel), and a significant decrease over time (figure 8, bottom panel). For anchovy, EOF1 captured 57.1% of the energy variability in spring, with a dominance of positive anomalies in the northwestern BoB (figure 7, top panel), and a significant increase over time (figure 7, bottom panel). In the surface layer (electronic supplementary material, figure A18), EOF1 explained 51.8% of the variance, with positive anomalies in the northwestern BoB and a significant increase over time. In the bottom layer (electronic supplementary material, figure A19), the EOF1 accounted for 44.1% of the variance, displaying similar spatial pattern as in the surface layer but without significant decrease over time. In autumn, the dominant mode explained 32.2% of the variance and revealed positive anomalies all along the shelf break (figure 8, top panel), with a significant increasing trend over time (figure 8, bottom panel). Regarding sprat, EOF1 explained 28.6 and 42.9% of energy variability in spring and autumn, respectively. In both seasons, positive anomalies were located in the northeastern BoB (figures 7c and 8c, top panels) with no significant trend over time (figures 7c and 8c, bottom panels). For horse mackerel, the primary mode of energy variability (EOF1, 39% of variance) in spring revealed widespread positive anomalies across the BoB (figure 7, top panel). A significant decreasing trend was observed (figure 7, bottom panel). In autumn, EOF1 explained 31.3% of the variance, with positive anomalies concentrated along the shelf break (figure 8, top panel), but with no clear temporal trend (figure 8, bottom panel). The variation in sprat and horse mackerel energy per depth strata was not analysed because they were almost exclusively distributed near the seabed. At the community level, EOF1 explained 45.9 and 29.5% of energy variability in spring and autumn, respectively. In both seasons, positive anomalies were concentrated in the northwestern BoB (figures 7e and 8e, top panels) with no significant trend in spring and a significant increase over time in autumn (figures 7e and 8e, bottom panels). In spring, while the surface (EOF1 explaining 32.3% of the variance, electronic supplementary material, figure A16) and bottom (EOF1 explaining 41.7% of the variance, electronic supplementary material, figure A17) layers exhibited similar spatial patterns, with positive anomalies in the northwestern BoB, a significant decreasing trend was observed only in the bottom layer.

#### 3.4.2. Temporal variations of the centres of gravity of prey energy

**Figure 9.**
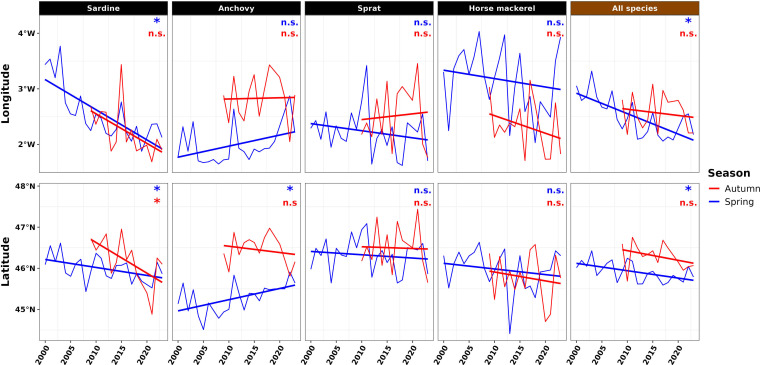
Temporal variation in the longitude and latitude of the centres of gravity at population (sardine, anchovy, sprat and horse mackerel) and community (all species) levels in the BoB in spring and autumn. Significance level: non-significant (*p*-value > 0.05, noted n.s.), significant (*p*-value < 0.05, noted *).

Time series of CGs’ latitude and longitude at population and community levels in spring and autumn are shown in figure 9. In spring, the latitude and longitude of the CG for sardine energy have significantly shifted southeastwards over time (figure 9). For anchovy, the latitude of the CG has significantly shifted northwards. No significant shift was observed for sprat and horse mackerel CGs over the past two decades. At the community level, the energy CG has also shifted southeastwards, driven by the sardine energy distribution. In autumn, no significant shift in CG latitude or longitude was detected, except for sardine energy, whose latitude shifted southwards.

## 4. Discussion

We analysed the energy seascapes of common dolphins and their temporal and spatial variations using georeferenced biomass data from two acoustic surveys (spring and autumn) in the BoB, combined with energy density measurements. The decrease of SPF prey length observed over two decades [43–45] has negatively impacted the individual prey energy/quality, a trend further exacerbated by the concurrent decline of their energy density. As a result, common dolphins now have access to roughly half the energy per individual prey compared with 20 years ago. Although total prey energy in the BoB has remained relatively stable over time, its spatial distribution has shifted closer to the coast in autumn, with marked interannual and species-specific variations. We will discuss how these changes in common dolphin energy seascape might have influenced their foraging behaviour and distribution, potentially affecting its population dynamics.

### 4.1. Smaller and less energetic prey

The energy density of SPF varies with length, especially for sardine and anchovy, showing notable seasonal differences [50]. Notably, sardines above 16 cm in spring and 20 cm in autumn (2014−2016) showed a decrease in ED, possibly due to reduced energy intake or increased metabolic costs [50]. Over the last 20 years, individual energy content of sardine and anchovy has significantly decreased in spring by factors of 2.5 and 2, respectively. This decrease was mainly driven by shrinking body sizes (sardine lost approx. 4 cm, anchovy approx. 2.5 cm). Sprat also showed a size decrease consistent with Mediterranean trends [66], while horse mackerel size remained stable. Autumn trends were less clear due to shorter time series. Sardine and anchovy individual ED values were higher in autumn than in spring, which was expected as both species are known to store energy from spring to autumn. Sprat and horse mackerel individual ED values were lower in autumn than in spring. Long-term declines in average prey energy were similar in both seasons. A significant decrease of sardine, anchovy and horse mackerel length in common dolphin stomachs was reported [36]. Two main effects influence individual prey energy: (i) body length, (ii) energy density and (iii) their interaction, since smaller fish store fewer lipids. Our results solely based on prey length probably underestimate energy loss, as ED itself has decreased over time, at least for sardines (canning industry data) and also probably for anchovy and horse mackerel [67]. Additionally, an increasing proportion of smaller, less energy-dense species like anchovy further reduces the energy of a random average prey. Longer ED time series for multiple species would be needed to assess more precisely the relative impacts of length and ED decrease on available prey energy. ED and lipid content are key indicators of prey quality for marine predators [68]. Environmental drivers such as temperature, nutrient supply and hydrodynamics influence phytoplankton and zooplankton nutritional value [69,70], directly affecting sardine and anchovy lipid reserves [71]. Prey size also plays a critical role in sardine condition, as smaller prey consumed by filter feeding require more energy to process than larger ones consumed by snapping [72]. Rising sea temperature further increases energy expenditure by elevating metabolic rates, exacerbating the challenge of building and maintaining energy reserves [73]. All these factors probably contributed to the decrease of common dolphin prey quality in the BoB over the last two decades.

### 4.2. Seasonal variation of energy seascapes

Seasonality strongly affects ED, especially for larger sardine and anchovy [50]. Seasonal ED changes are moderate for smaller fish but more pronounced in larger individuals that store energy for overwintering [74]. Canning industry data indicate that the long-term decline observed in sardine ED mainly occurred in autumn, while spring values remained relatively stable over the last two decades. This probably reflects seasonal changes in fish physiology. In fact, most energy reserves have been depleted in spring after overwintering [75], which limits the effect of ED decline. Energy decrease is then mainly caused by fish length decline in spring, as shown by our simulation results. In autumn, the decrease in prey energy results from both fish shrinking and decreasing ED. Between 2009 and 2023, total energy from SPF potentially available to common dolphins was higher in spring than in autumn, reflecting the variations of sardine, which was the main energy provider. This result was not expected, as sardines grow and accumulate reserves between spring and autumn, their population energy should be higher in autumn than in spring. We then assume that the observed decrease in total/sardine energy between spring and autumn may result from a reduction of the sardine abundance in the BoB in autumn, resulting from several, mutually non-exclusive, processes. First, sardines may migrate outside the BoB in autumn, as suggested by earlier population studies [76] and recent genetic analyses [77]. Second, the observation of larger sardines in poor condition in spring [51] can have consequences on post-spawning mortality and the decrease in abundance, though unlikely to fully explain it. As most of sardine catches occur in summer in the BoB [42], fishing activities probably contribute to reducing sardine abundance between spring and autumn. Finally, despite similar protocols in PELGAS (spring) and JUVENA (autumn) surveys [58], differences in target species or fish accessibility (e.g. very coastal sardines not accessible to survey vessels) may partly explain seasonal discrepancies observed in sardine abundance/energy.

Energy seascapes integrate prey biomass, individual size and energy content, to provide a more accurate picture of the spatial heterogeneity of prey quality. On average, sardine energy was mainly distributed near the coast, with a secondary offshore component in spring, and concentrated closer to the coastline in autumn, with almost no sardine energy detected beyond the 100 m isobath. Anchovy energy shifted from the main spawning area in southeast BoB in spring to the feeding grounds in the northeast in autumn on average [49,78,79]. These seasonal migrations probably aim at maximizing individuals’ condition and fitness [80]. Our results suggest a clear offshore-to-onshore migration for horse mackerel, while sprat maintains a very coastal distribution year-round, with a slight offshore expansion in autumn. At the community level, energy concentrated near the coast in autumn, particularly along the northern BoB. The spatial overlap between sardine and anchovy energy was hence lower in spring and increased in autumn, as both species shared the same coastal feeding grounds. Since these two species are the primary prey of common dolphins [17,34,35], the coastal areas where they co-occurred probably represent highly favourable foraging habitats for dolphins. Seasonal changes in prey distribution were probably driven by environmental factors such as sea surface temperature, chlorophyll-a concentration, stratification and streamflow regimes [81–83], which influence SPF zooplanktonic prey availability and thus SPF distribution. Garcia-Soto and Pingree [84] showed that phytoplankton blooms occurred regularly in the offshore region of the BoB in spring, but not in autumn, possibly explaining why SPF energy was more coastal in autumn. In turn, common dolphins probably forage closer to the coast in autumn and near the shelf break in spring [85] to follow the seasonal changes evidenced here in their prey energy seascape. Several studies have documented the distributions of SPF single species in the BoB in spring [61] and in autumn [86], and assessed sardine seasonal distribution [87]. To our knowledge, our study provides the first assessment of the distribution of several SPF species in spring and autumn in the BoB. Despite the existence of seasonal surveys, our understanding of SPF feeding and spawning migrations in this region remains limited [88], even though such processes are well described in other marine ecosystems [48,81,83]. By analysing two large-scale acoustic surveys conducted in spring and autumn, our study offers new insights into seasonal shifts in species distributions and associated energy dynamics.

### 4.3. Evolution of common dolphin energy seascapes over the last two decades

Space-time analyses based on empirical orthogonal functions and centres of gravity revealed significant interannual variations in common dolphin energy seascapes, with marked differences between prey species and seasons. While sardine and anchovy energy distribution showed significant spatio-temporal shifts, sprat and horse mackerel ones remained relatively stable in both spring and autumn. Sardine energy distribution has progressively shifted closer to the coast during both seasons, with a marked decline of the offshore secondary energy hotspot, resulting from the disappearance of larger sardine in northwestern areas. This pattern echoes previous findings on sardine egg distribution [46], which showed a reduction in the use of the offshore spawning habitat in northwestern BoB as the population’s size structure shifted towards smaller individuals. Conversely, anchovy energy has expanded from core southern distribution areas towards the northwestern areas deserted by larger sardine in spring and along the shelf break in autumn in the BoB. This seasonal shift in energy distribution is expected to intensify with climate change [89].

In spring, the opposite space-time trends followed by sardine and anchovy energy in northwestern BoB may be linked to changes in the availability of the distinct mesozooplankton community composed of large individuals observed in this area [90,91]. In fact, in recent years, these large zooplankton have undergone a notable reduction in size, despite stable biomass [71], potentially reducing their quality at the BoB scale. This structural change probably results from three concurrent processes: (i) an increase in the abundance of small individuals, (ii) a reduction in the average size of small zooplankton, and (iii) a decline in the average size of large zooplankton. These patterns are consistent with a bottom-up effect, whereby changes in prey size and quality propagate through the food web. Similar dynamics have been documented in Namibia, where a rise in small copepods was linked to reduced fish size and shifts in their spatial distribution [92]. This suggests that the observed decline in small pelagic fish condition in the Bay of Biscay may not be driven by reduced zooplankton biomass, but rather by a qualitative shift in zooplankton community structure.

These dynamics probably reflect a combination of biotic interactions and broader physico-chemical changes driven by climate change, making it difficult to fully disentangle the mechanisms behind the inverse spatio-temporal patterns observed between the two species.

Long-term compensatory dynamics are a recognized basis of community stability and ecosystem resilience [93,94], whereby the decline of one species can be balanced by the rise of another. Anchovy expansion has here compensated for the local depletion of sardine energy. Energy hence remained stable at the community level, through compensation effect [94]. However, while overall prey energy available for dolphins remained stable, the individual prey quality of the replacing species was lower. Long-term consequences on predator energy intake hence remain uncertain. These results mirror those reported for the BoB springtime zooplankton community, whose total biomass has remained stable over time despite a marked decrease in individual size, which was compensated by an increase in the abundance of smaller individuals [71]. For high-energy-expenditure predators like common dolphins, the marked decline in prey energy represents a major challenge to sustaining daily energetic needs [32]. With average prey now estimated to be 2.2 times less energetic than two decades ago, dolphins must capture and consume many more individuals to meet their metabolic requirements. This not only increases the time and effort invested in foraging, but also makes success more dependent on prey encounter rates, which are inherently probabilistic. As highlighted in [95], this element of ‘luck in finding food’ can play a decisive role, particularly when prey fields are less profitable.

Prey distribution, abundance and quality alone do not fully determine a predator foraging success [96]. Because average prey concentrations in open waters are generally too low to meet predator energy requirements [96,97], the ability to locate and exploit prey patches is critical for foraging success. Key patch characteristics such as depth, density, size and spatial arrangement strongly influence predator energy intake and behaviour [98]. In our study, we observed a decline in sardine energy offshore below 30 m depth, while the increase in anchovy energy was mostly concentrated in the upper surface layers in the northern BoB. Although common dolphins are capable of diving to depths of 200 m [99], they typically forage between the surface and 50 m depth [100,101], where small pelagic fish are most abundant and energy expenditure is minimized [102]. This shift towards more surface-layer prey could, in principle, benefit common dolphins by increasing prey accessibility. However, because these surface resources are largely composed of smaller, less energy-rich anchovies replacing sardines, the net energetic benefit for dolphins may be limited. Beyond depth, other patch attributes—including size, density, shape and spacing—can strongly influence predator foraging strategies [98]. In the BoB, however, prey patch characteristics appear relatively similar among the main small pelagic fish species [103], especially compared with the contrasting prey types (e.g. krill swarms vs. juvenile pollock schools) studied by Benoit-Bird et al. [98]. Our data further suggest that patch structure has remained relatively stable over time. This indicates that long-term changes in prey size, energy content and distribution are more likely to explain the observed modifications in dolphin foraging habitats than shifts in patch structure. Nevertheless, a more detailed investigation of the spatio-temporal variability of prey patch characteristics would be valuable for future research, in a context where prey patch characteristics would become even more critical for common dolphins, as they may compensate for lower per-prey energetic value.

Such changes could ultimately impact top predators like the common dolphin, through bottom-up control and cascading effects propagating throughout the pelagic food web of the BoB. The potential consequences of a decline in prey energy for common dolphins are concerning. In the Celtic Seas, declining body condition (as measured by ventral blubber thickness) and increased starvation-related strandings have already been documented [104]. Given that prey quality is often more critical than prey quantity for marine mammals [15,32,105], similar effects may be occurring in the BoB. While our analysis focused on common dolphin prey, the observed shifts in prey energy seascapes could also have implications for other top predators in the BoB such as seabirds, piscivorous fishes or other marine mammals. Our approach could be applied to these other species provided that the potential diets are adjusted before building the seascape. Our approach could likewise be applied to other ecosystems facing similar challenges, such as the Benguela upwelling system, where changes in zooplankton abundance and community composition have altered the size and distribution of small pelagic fish, with cascading effects on top predators such as seabirds [92,106,107]. Such bottom-up alterations of prey fields, and their consequences for predator foraging, are expected to become more frequent under climate change, with the potential to severely affect top predators through trophic amplification mechanisms [10,108].

## 5. Conclusion

Using an integrative approach, we revealed substantial changes in the common dolphin energy seascape in the BoB. Individual prey energy has significantly declined, driven by a combination of shrinking fish size and decreasing energy density. Consequently, dolphins have to consume a greater number of smaller, less energetic prey individuals to maintain their energy intake. Changes in prey quality and distribution may have already affected dolphin foraging strategies, potentially increasing their spatial overlap and interactions with fisheries [109]. Current annual dolphin by-catch has been estimated between 5000 and 10 000 individuals [110], raising serious concerns for the population’s long-term viability [111,112]. While the energy seascape approach provides critical insights into prey availability, a more complete picture of the foraging seascape should include the energetic costs of hunting, capturing and processing prey [113]. However, acquiring such data remains challenging, particularly due to the logistical and ethical constraints of studying common dolphins in the wild. In parallel, more knowledge on common dolphin feeding ecology and physiology would be needed to fully understand how changes in prey seascape may have affected dolphins’ fitness.

## Data Availability

PELGAS data: [114]. JUVENA data: [115]. Energy density data: [59]. Supplementary material is available online: [116].
